# Fatal Case of Fungemia by *Wickerhamomyces anomalus* in a Pediatric Patient Diagnosed in a Teaching Hospital from Brazil

**DOI:** 10.3390/jof6030147

**Published:** 2020-08-25

**Authors:** Vitor Rodrigues Dutra, Leonardo Francisco Silva, Adriana Nazaré Miziara Oliveira, Emília Freitas Beirigo, Vanessa Mello Arthur, Raíssa Bernardes da Silva, Thatiana Bragine Ferreira, Leonardo Andrade-Silva, Marcos Vinícius Silva, Fernanda Machado Fonseca, Mario León Silva-Vergara, Kennio Ferreira-Paim

**Affiliations:** 1 Department of Microbiology, Immunology and Parasitology, Federal University of Triangulo Mineiro, Uberaba 38015-050, Brazil; vitordutrarod@gmail.com (V.R.D.); leonardosilva1972@hotmail.com (L.F.S.); emiliabeirigo@hotmail.com (E.F.B.); vanessa.mello1703@hotmail.com (V.M.A.); raissabsil@hotmail.com (R.B.d.S.); marcos.silva@uftm.edu.br (M.V.S.); 2 Department of Cardiology, Federal University of Triangulo Mineiro, Uberaba 38025-440, Brazil; adrinami@terra.com.br; 3 Department of Infectious Diseases, Federal University of Triangulo Mineiro, Uberaba 38025-440, Brazil; tatibragin@yahoo.com.br (T.B.F.); leonardoeuripedes@gmail.com (L.A.-S.); marioleon1956@gmail.com (M.L.S.-V.); 4 Department of Biomedicine, Federal University of Triangulo Mineiro, Uberaba 38025-350, Brazil; fernanda.fonseca@uftm.edu.br

**Keywords:** *Wickerhamomyces anomalus*, pediatrics, fungal diseases, emerging diseases, molecular epidemiology, ITS sequencing, case report

## Abstract

In recent decades, emerging fungal infections have changed the clinical mycology scenario as a consequence of the advances in medical diagnostics and therapeutic procedures, long hospitalization times, and the growing number of individuals with debilitating chronic diseases and impaired immune systems. This report presents a 19 months old Brazilian female patient who developed a severe fungal sepsis by an uncommon yeast. She was admitted at the intensive care unit with severe pneumonia, bronchopulmonary dysplasia, and weight-for-age z score of less than −2. She remained more than 30 days in the intensive care unit where she had a femoral venous catheter placement, enteral nutrition, broad-spectrum antibiotic therapy, and prophylaxis with fluconazole. Moreover, pericardiocentesis was performed due to cardiac tamponade. She had a previous history of prematurity, cardiac surgery due to patent ductus arteriosus, and a long period of hospital stay. Despite the antifungal prophylaxis, two yeast isolates were recovered from blood and then identified by classical mycological methods and internal transcribed spacer (ITS) sequencing as *Wickerhamomyces anomalus*. Both isolates exhibited susceptibility to amphotericin B, ketoconazole, itraconazole, voriconazole, and fluconazole. Her clinical state worsened, presenting anasarca, epistaxis, and hemorrhagic suffusions in the mouth, sclera, oliguria, and bradycardia. Two days after the first positive culture, she presented a gradual reduction of the white blood cells count, with severe leukopenia and neutropenia. She died five days after.

## 1. Introduction

Fungemia is one of the most common causes of morbidity and mortality in hospitalized patients. Most infections are related to *Candida* species, although other emergent fungi are becoming more common as a consequence of the advances in medical diagnostics and therapeutic procedures, longer hospitalization times, growing number of individuals with debilitating chronic diseases, and impaired immune system [1,2,3].

The yeast *Wickerhamomyces anomalus*, formerly known as *Pichia anomala*, *Hansenula anomala*, and *Candida pelliculosa*, is often found in soil, plants, fruits, and grain products. It has been well studied as a bio preservation agent in many different post-harvest systems due to its antimicrobial properties, including the production of killer toxins and volatile metabolites [4,5].

*Wickerhamomyces anomalus* infections are usually more prevalent in infants, especially in those hospitalized in pediatric intensive care units (PICU) than in adults [6,7]. The higher prevalence in this population is related with the history of prematurity and low birth weight [8]. The use of a central venous catheter, total parenteral nutrition, and immunosuppression are also some of the main risk factors associated with its recovery from the blood in adults and children [2,7,9,10].

Nosocomial infections caused by *W. anomalus* in adults and pediatric patients have been reported around the world with a mortality rate up to 38% and 42%, respectively [8,11,12]. The first published outbreak in PICU described five neonates with fungemia, two with fungemia and ventriculitis, and one with ventriculitis. All of them presented low birth weight and prematurity as risk factors with a mortality rate of 25% [13]. In Brazil, 17 out of 1046 children admitted in the PICU from October 2002 to January 2004 presented fungemia by *W. anomalus*. The main risk factors were congenital malformations and neoplastic diseases with an overall mortality rate of 41.2% [6].

Even though this yeast is considered an opportunistic agent, isolated cases and sporadic outbreaks with high mortality have been reported in PICU, which emphasizes the relevance to considering this fungus within the diagnostic possibilities [14,15]. Here, we present a fatal case of fungemia due to *W. anomalus* in a pediatric patient admitted in a Brazilian tertiary hospital.

## 2. Case Report

A 19 month old Brazilian female was admitted at the emergency unit of a teaching hospital with fever, cough, irritability, and dyspnea. Her mother said that the child was on one week’s use of amoxicillin and bronchodilator drug.

**Figure 1:**
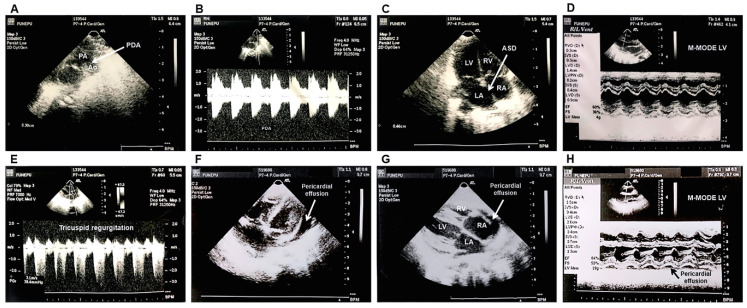
Echocardiographic assessment showing the presence of the patent ductus arteriosus (PDA) and interatrial communication 14 days after birth (**A**–**E**) and pericardial effusion in the last hospitalization of the 19 month old girl (**F**–**H**): (**A**,**B**) Transversal view indicating the PDA of 3.8 mm and Doppler showing a systodiastolic flow through the PDA, respectively. (**C**,**E**) Apical view indicating atrial septal defect (ASD) and tricuspid regurgitation with pulmonary hypertension respectively. (**D**) M-mode echocardiogram showing preserved left ventricular function. (**F**,**G**) Apical and subcostal view respectively indicating the pericardial effusion. (**H**) Transversal view showing a pericardial effusion of 7.0 mm and preserved left ventricular function. Legend: Ao: aorta. ASD: atrial septal defect. LA: left atrium. LV: left ventricle. PA: pulmonary artery. PDA: patent ductus arteriosus. RA: right atrium. RV: right ventricle.

She was born by C-section at 29 weeks and six days in the Obstetric Emergency Unit of the same institution and was immediately transferred to the PICU and intubated because of her critical clinical state. Her birth weight was 1020 g. By day 13, she presented a bloodstream infection due to *Candida albicans*, which was successfully treated with amphotericin B (AMB). The heart auscultation showed a presence of a continuous murmur and the echocardiography identified the patent ductus arteriosus (PDA) and tricuspid regurgitation, which led to a surgical correction (Figure 1A–E). An iatrogenic chylothorax was diagnosed and during the procedure to repair it, the child presented cardiopulmonary arrest and the surgery was stopped. Then, a thoracentesis was performed with a placement of a chest tube, which remained for three months. A few days after the thoracentesis, a positive urine culture for *C. albicans* was evidenced and she received amphotericin B for 10 days. By day 30, she presented a positive catheter tip culture for *Pseudomonas aeruginosa* with a negative blood culture, successfully treated with the catheter removal. By day 43, *Candida albicans* and coagulase-negative staphylococci bacteria were recovered from blood and treated with AMB and penicillin, respectively. Due to the presence of cervical edema, an echocardiogram was performed and a superior vena cava thrombosis was evidenced. She was heparinized for three and a half months. After seven months, she was discharged from the PICU. However, the child was admitted in the same hospital in two different occasions due to fungal and bacterial infections.

At admission she was afebrile and weighed 7.0 kg. Pulmonary auscultation: presence of bibasilar crackles and rhonchus. Heart: mild systolic murmur. Abdomen: flaccid and distended. Liver: 3 cm below costal margin. Chest X-ray evidenced cardiomegaly and a right middle lobe consolidation. The next day, she presented cyanosis, anasarca, chest in-drawing, and worsening of her general clinical state. A moderate pericardial effusion with hemodynamic repercussion was evidenced at echocardiogram (Figure 1F–H). She was admitted to the PICU, intubated, and treated with vasoactive drugs, diuretics, fluconazole, ceftriaxone, and oxacillin due to sepsis hypothesis. One week after, the antibiotics scheme was replaced by vancomycin plus cefepime. A pericardiocentesis was performed and the pericardial fluid analysis showed pH 8.0, density 1.030, glucose 60.8 mg/dL, lactate dehydrogenase 1362.0 U/L, amylase 5.0 U/L, and proteins 5.19 g/dL. Gram, culture for bacteria and fungus, and Ziehl-Neelsen staining were negative. Over the next three weeks, her clinical condition remained unchanged. Despite 12 days on fluconazole (FLZ) prophylaxis (25.0 mg/kg/day), a positive culture for *Candida* sp. was identified. A second blood culture six days later identified the same microorganism. After 14 days on fluconazole (25.0 mg/kg/day) use, a maintenance dose (6.0–12.0 mg/kg/day) was prescribed and amphotericin B (attack doses: 0.5, 1.5, 3.5, 4.5, 5.5, and 11.5 mg/kg/day) implemented. Two days after the first positive culture, there were a gradual reduction on the hemoglobin level and white blood cells (WBC) count, with severe leukopenia and neutropenia for which she received granulokine and blood transfusion. In a period of 10 days, the WBC count reduced from 10,990 cells/mm^3^ (bands: 1429 cells/mm^3^, neutrophils: 8571 cells/mm^3^, eosinophils: 220 cells/mm^3^, lymphocytes: 440 cells/mm^3^, and monocytes: 330 cells/mm^3^) to 180 cells/mm^3^ (neutrophils: 14 cells/mm^3^ and lymphocytes: 166 cells/mm^3^). Her clinical state worsened presenting anasarca, epistaxis and hemorrhagic suffusions in the mouth and sclera, oliguria, and bradycardia. She died five days after.

### Phenotypic, Molecular Characterization, and Antifungal Susceptibility Tests

The blood samples were collected using the BACTEC system (Difco^TM^, Sparks, Baltimore, MD, USA) and processed through the BACTEC 9240 (Becton, Dickinson, and Company, Sparks, MD, USA) automated culture system. Presence of yeast cells was confirmed by Gram staining of the positive vials. Blood was subcultured in CHROMAgar *Candida* medium (Becton, Dickinson, and Company, Sparks, Baltimore, MD, USA) for 24–48 h at 37 °C yielding small pink colonies. The germ tube test was negative. As the first line of identification, colonies were subjected to VITEK^®^ 2 (Biomeriux, Marcy l’Etoile, Lyon, France) and this identified both isolates (UFTM 08.01 and UFTM 08.02) as *C. pelliculosa*. Antifungal susceptibility tests to AMB, ketoconazole (KET), itraconazole (ITZ), voriconazole (VOR), and FLZ were performed according to CLSI M27A3 [16]. Both isolates were susceptible to all antifungal tested. The Minimal Inhibitory Concentration, MIC (µg/mL), range were as follows: 0.12–0.25 for AMB, 0.12 for KET and ITZ, 0.12–0.25 for VOR, and 1.0–2.0 for FLZ.

**Figure 2:**
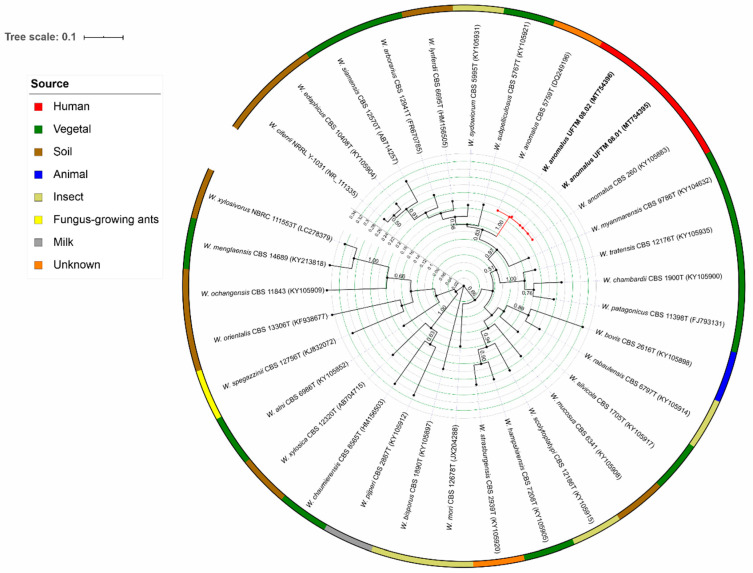
Maximum likelihood tree of the *Wickerhamomyces* species using the sequences of the internal transcribed spacer (ITS) region. The tree with the highest log likelihood (−7874.49) drawn to scale with branch lengths measuring the number of substitutions per site is shown. Bootstrap values >50% based on 1000 replicates are presented close to the branches. The analysis involved 33 nucleotide sequences with 650 positions revealing that the two isolates of the present study (highlighted in bold) were clustered in the *Wickerhamomyces anomalus* clade, highlighted in red in the tree. The isolates are described according to the species name, followed by strain number, and GenBank number. The strip colors surrounding the tree presents the source of recovering of each strain, and are described as follows: red: human, green: vegetal, brown: soil, blue: animal, beige: insect, yellow: fungus-growing ants, grey: milk, orange: unknown.

Genomic DNA, internal transcribed spacer (ITS) region amplification and sequencing were performed as previously described [17,18]. The Basic Local Alignment Search Tool (BLAST) search revealed that the isolates showed 99% similarity with CBS 260 type strain of *W. anomalus*. The sequences of the herein described isolates were compared with the ITS sequences of different species of *Wickerhamomyces* retrieved from CBS-KNAW culture collection, NRRL Culture Collection, and NBRC Culture Collection. The sequences were aligned using the Muscle algorithm in Mega X [19,20]. The best substitution model, K80, was calculated in jModelTest v. 2.1.10 [21]. The Maximum Likelihood tree was inferred and visualized using the Interactive Tree of Life. This analysis confirmed the identification of both isolates as belonging to *W. anomalus* clade, composed of the type strain CBS 5758 and CBS 260, the last one also recovered from blood (Figure 2).

## 3. Discussion

Invasive fungal infections in pediatric patients are usually associated with high morbidity and mortality. The most common site of infection is the blood and the *Candida* species are by far the most common pathogens recovered [22]. *Wickerhamomyces anomalus* is one of the most pre-eminent species in the *Wickerhamomyces* genus responsible for fungemia. This is of particular importance in the PICU, where it has been associated with some outbreaks of clonal origin [14,23].

In this report, we describe a 19 months old girl with several underlying conditions associated with prematurity, long hospital stay, several diagnostic and therapeutic procedures, malnutrition, and a broad-spectrum antibiotic use. Most of these factors are strongly related to *Candida* infections. The malnutrition has been considered one of the most important factors associated with fungemia because of its direct and indirect impact on the immune response [24], and it can be evidenced by the very low weight identified in the child of the present case who developed several infections events during her long hospital stay. Thus, all of these underlying conditions have probably contributed with the poor outcome of the case herein described.

Since the isolates were recovered in two different occasions from blood with three negative cultures of catheter tips, it would be less probable that the infection had occurred via catheter. Moreover, the pericardiocentesis procedure was performed 28 days before the first fungemia episode, which excludes this fact as the probable source of infection.

Other sources of infection with rare yeasts have been described. Recently, *Kluyveromyces marxianus* (formerly *Candida kefyr*) was recovered from the blood of an immunocompromised hematological patient with mucositis and history of frequent ingestion of dairy products [25]. Another report describes *Saccharomyces cerevisiae* causing fungemia in two COVID-19 patients receiving probiotic preparations with this yeast [26]. *Wickerhamomyces myanmarensis*, a close relative of *W. anomalus*, was recovered from the blood and central venous catheter of a 5.5-year-old boy and the episode was associated with consumption of honey, use of proton pump inhibitor, and gut mucosa inflammation [1]. The patient herein reported was receiving nasoenteral feeding at the time of the yeast isolation. *W. anomalus* has already been described in the human gut microbiota and certain conditions such as intestinal inflammation/infection allow for the predisposition to gut translocation which could have happened with this case [27,28].

The emergence of rare fungal species with different antifungal susceptibility profiles causing infections in patients with underlying conditions remarks the relevance of their correct identification aiming to improve the therapeutic decision and outcome. Phenotypic identification is insufficient to accurately identify close related species, which highlights the pivotal importance of molecular techniques such as Polymerase chain reaction-restriction fragment length polymorphism (PCR-RFLP), Matrix-Assisted Laser Desorption/Ionization Time-Of-Flight Mass Spectrometry (MALDI-TOF MS), and DNA sequencing in the clinical laboratory [2,29,30]. The phylogenetic analysis of the ITS sequencing obtained from both isolates confirmed that they belong to the *W. anomalus* clade. Moving towards sequencing of all clinically uncommon fungal isolates would be highly desired in resource-limited settings where automated yeast identification can represent the final step fungal identification.

The results of the antifungal susceptibility tests of both isolates herein described are in agreement with the scarce availability of data on *W. anomalus* showing a high susceptibility profile [2,14]. Despite this susceptibility to most antifungals drugs, antifungal resistance to voriconazole was already described in Brazil, and it highlights the importance of monitoring the occurrence of this species in the PICU [14].

The availability of new therapies for malignant, inflammatory, and debilitating chronic diseases, in addition to the increase of diagnostic and therapeutic procedures, have increased risk for emerging pathogens, including rare fungal species that have changed the global scenario of the clinical mycology. Thus, the description of uncommon mycoses cases is important to alert the clinical and mycology communities and to reinforce the implementation of molecular diagnostic tools in clinical laboratories.
